# Reliability of Two Diameters Method in Determining Acute Infarct Size. Validation as New Imaging Biomarker

**DOI:** 10.1371/journal.pone.0140065

**Published:** 2015-10-08

**Authors:** Jochen B. Fiebach, Jonas D. Stief, Ramanan Ganeshan, Benjamin Hotter, Ann-Christin Ostwaldt, Christian H. Nolte, Kersten Villringer

**Affiliations:** 1 Center for Stroke Research Berlin (CSB), Department of Neurology, Charité Universitätsmedizin Berlin, Hindenburgdamm 30, 12200, Berlin, Germany; 2 Department of Radiology, Charité—Universitätsmedizin Berlin, Campus Virchow-Klinikum, Augustenburger Platz 1, 13353, Berlin, Germany; 3 Massachusetts General Hospital, Center for Human Genetic Research, 185 Cambridge Street, Boston, MA, 02114, United States of America; Henry Ford Health System, UNITED STATES

## Abstract

**Background:**

In order to select patients most likely to benefit for thrombolysis and to predict patient outcome in acute ischemic stroke, the volumetric assessment of the infarcted tissue is used. However, infarct volume estimation on Diffusion weighted imaging (DWI) has moderate interrater variability despite the excellent contrast between ischemic lesion and healthy tissue. In this study, we compared volumetric measurements of DWI hyperintensity to a simple maximum orthogonal diameter approach to identify thresholds indicating infarct size >70 ml and >100 ml.

**Methods:**

Patients presenting with ischemic stroke with an NIHSS of ≥ 8 were examined with stroke MRI within 24 h after symptom onset. For assessment of the orthogonal DWI lesion diameters (od-values) the image with the largest lesion appearance was chosen. The maximal diameter of the lesion was determined and a second diameter was measured perpendicular. Both diameters were multiplied. Od-values were compared to volumetric measurement and od-value thresholds identifying a lesion size of > 70 ml and > 100 ml were determined. In a selected dataset with an even distribution of lesion sizes we compared the results of the od value thresholds with results of the ABC/2 and estimations of lesion volumes made by two resident physicians.

**Results:**

For 108 included patients (53 female, mean age 71.36 years) with a median infarct volume of 13.4 ml we found an excellent correlation between volumetric measures and od-values (r2 = 0.951). Infarct volume >100 ml corresponds to an od-value cut off of 42; > 70 ml corresponds to an od-value of 32. In the compiled dataset (n = 50) od-value thresholds identified infarcts > 100 ml / > 70 ml with a sensitivity of 90%/ 93% and with a specificity of 98%/ 89%. The od-value offered a higher accuracy in identifying large infarctions compared to both visual estimations and the ABC/2 method.

**Conclusion:**

The simple od-value enables identification of large DWI lesions in acute stroke. The cutoff of 42 is useful to identify large infarctions with volume larger than 100 ml. Further studies can analyze the therapeutic utility of this new method.

**Trail Registration:**

ClinicalTrials.org NCT00715533

## Background and Purpose

In acute ischemic stroke, volume assessment of the infarcted tissue has been used to select patients most likely to benefit for thrombolysis and to predict patient outcome [1]. Diffusion-weighted imaging (DWI) enables an excellent infarct visualization even for readers with limited clinical experience [2]. Despite the intense signal of infarcted tissue on DWI, size estimation has limited agreement rates. In the time-sensitive setting of stroke, there is a need for a rapid and easy method for infarct size assessment. Sims and coworkers investigated which geometric shape—calculated on grounds of infarct diameters—best met volumetric results [3]; their so-called ABC/2 formula led to reproducible and accurate estimation of planimetric results in 63 patients with a slope of 1.16 in linear regression. However, discrepant observations were reported by Pedraza and coworkers based on DWI from 86 patients. With the ABC/2 technique they observed a 62% overestimation of acute DWI lesions [4]. Thus, the accurate estimation of infarct volume using ABC/2 remains unclear. Since only extended infarction needs to be ruled out before initiation of acute therapy [1, 5], we tested whether a further simplification of lesion size estimation applying orthogonal DWI lesion diameters (od-value) sufficiently enables identification of large infarcts.

## Methods

### Patients and image acquisition

**Fig 1:**
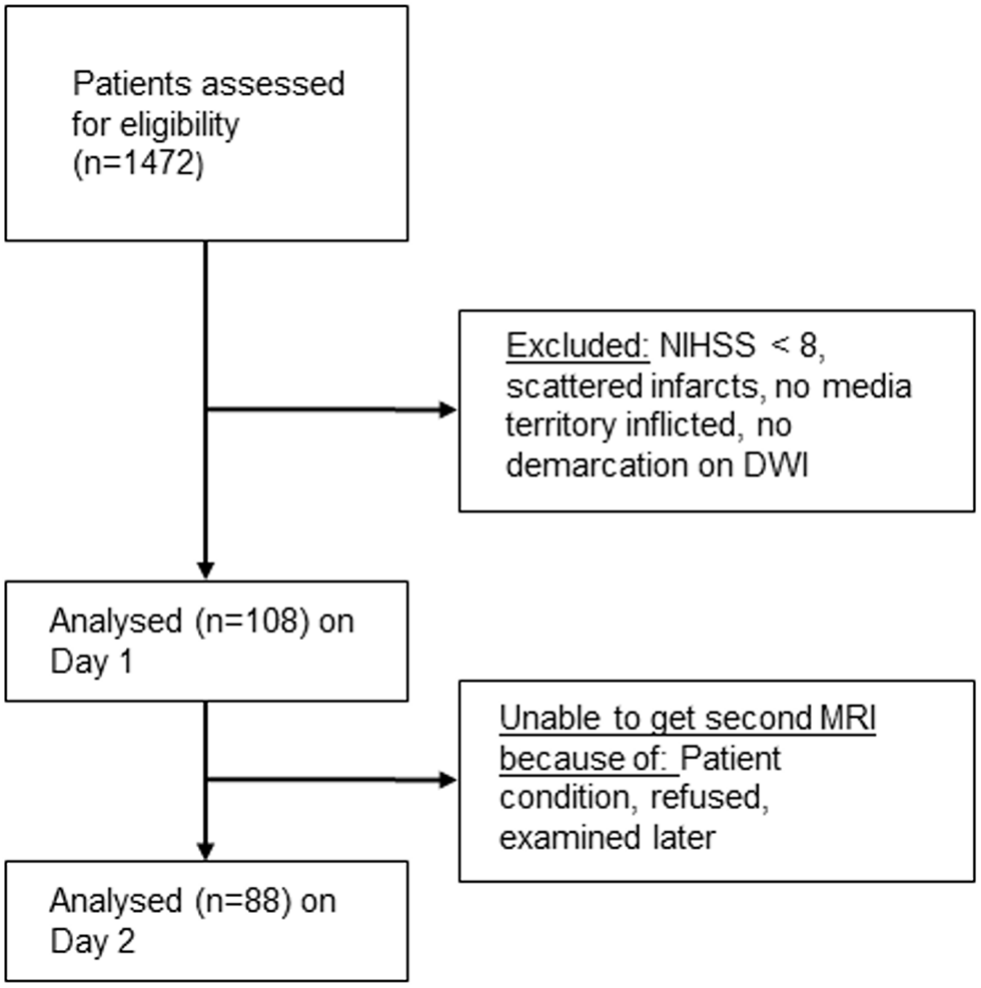
Flow diagram. Patient selection criteria for this study.

Patients presenting with clinical stroke severity of at least 8 on the National Institutes of Health Stroke Scale (NIHSS) score and ischemic lesions on DWI in the territory of the middle cerebral artery qualified for this substudy of the 1000+ program (clinicaltrials.org NCT00715533). The authors confirm that all ongoing and related trials for this study are registered. A Flow diagram shows the Patient selection criteria for this substudy (Fig 1). The total results of this study are not prepared for publication yet. The protocol paper was published [6]. Recruitment started in October 2008 and ended in June 2013. The ethics committee approved study design and trail protocol before the trail began. All patients gave written informed consent; see S1 Protocol and S1 STARD Checklist in the supporting information.

MRI examinations were performed on a 3 Tesla Siemens Tim Trio within 24h from symptom onset and if possible on day 2 after stroke. Our stroke MRI protocol [6] includes high resolution diffusion weighted imaging conducted as DTI with 6 directions at b = 1000 s/mm*2, TE = 93 ms, TR = 8,000 ms, 2.5 mm slice thickness.

### Ethics statement

Written informed consent was obtained from all patients. The study design (1000+ study) was approved by the ethics committee of the Charité—Universitätsmedizin, Berlin (EA4/026/08).

### Determination of od-values and volumetric measures

**Fig 2:**
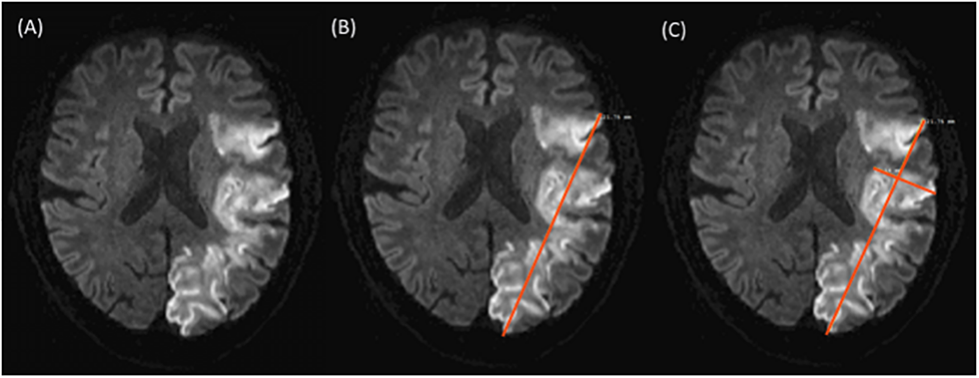
Illustration of the od-value calculation. (A) Shows the DWI slice with the largest lesion diameter. (B) Demonstrates how the maximal diameter of the lesion was measured on this slice. (C) Shows how a second perpendicular diameter was measured, again choosing the maximum length. In this infarct of 95 ml volume, the multiplication of the two values results in an od-value of 41.53.

Lesion volumes on DWI were delineated manually using MRIcro 1.40 (Chris Rorden, USA). For od-value assessment the image demonstrating the largest lesion diameter was chosen. The maximal diameter of the lesion was determined on this slice and a second diameter was measured perpendicular, again choosing the maximum length (Fig 2). Multiplication of both values led to the od-value.

### Lesion size estimation in a compiled dataset

Since the standard for judging lesion size in the acute setting is visual inspection we compared whether lesion sizes estimated visually are comparable to the od-values. Since infarct sizes in our cohort were relatively small, we compiled a subset of DWI datasets (n = 50) containing 10 cases each for the lesion volume categories: > 100 ml, 100–60 ml, 60–40 ml, 40–20 ml and < 20 ml. Two resident physicians (RG and BH)—both with more than 3 years of clinical experience in stroke imaging—independently assessed the DWI datasets presented in a random order and judged lesion volumes. Both provided lesion volume estimation blinded to individual patients’ clinical status.

For comparison, also lesion volumes with the ABC/2 method were determined in the compiled dataset (od-values multiplied with the number of positive slices and slice thickness, divided by 2).

### Statistics

Statistics were performed using IBM SPSS (version 16). We calculated a Spearman’s correlation between the following volumetric assessments: od-values, ABC/2 values, and visual lesion size estimation (of the two raters). Od-value thresholds indicating infarct size >100 ml and > 70 ml were determined by generating ROC-curves and using the Yourden-Index. Bland-Altman plots were generated by using GraphPad Prism 5.

Sensitivity and specificity for predicting infarct sizes > 100 and > 70 ml was determined for the od-values as well as for the visual lesion size estimations and the ABC/2 method for the compiled dataset. A level of p ≤ 0.05 was considered significant.

## Results

### Comparison of the od-value with the volumetric measurements of the whole dataset

On day one 108 patients meeting the inclusion criteria were examined with a median delay of 133 min from symptom onset (IQR 65–474 min). Of these patients, 68 (63.0%) were scanned within the 4.5 h time window. Gender distribution was 56 male to 53 female, mean age was 71.36 years. Median stroke severity was 11 (IQR 9–16) on the NIHSS, 57 of the patients received rtPA and 2 endovascular treatment. On day 1, five (4.6%) patients presented with an infarct size of > 100 ml and seven (6.5%) with > 70 ml. Median infarct volume was 13.4 ml (range 0.1–273.8 ml) which corresponds to a median od-value of 7.36 (range 0.01–76.7). Regression of lesion volumes to od-values is expressed by the equation:

$$volume=2.13+0.947(a\;x\;b)+0.031{\left(a\;x\;b\right)}^{2}$$

Spearman correlation between volume and od-value was 0.951 (p < 0.01) for the baseline examination. Based on baseline DWI the od-value threshold for infarcts > 100 ml was 42 and for infarcts > 70 ml was it 32.

DWI datasets from day 2 after stroke were assessed for 88 patients. Two patients with extended infarctions at baseline were unable to undergo a second MRI examination; others refused or were examined later. On day 2, infarct volume > 100 ml and > 70 ml was found in 6 (6.8%) and 10 (11.4%) of the patients, respectively. Median DWI infarct volume was 9.0 ml ranging from 0.03 to 170.7 ml. Median od-value was 5.51 ranging from 0.06 to 54.54. Spearman correlation between volume and od-value was 0.978 (p < 0.01) at day 2.

**Fig 3:**
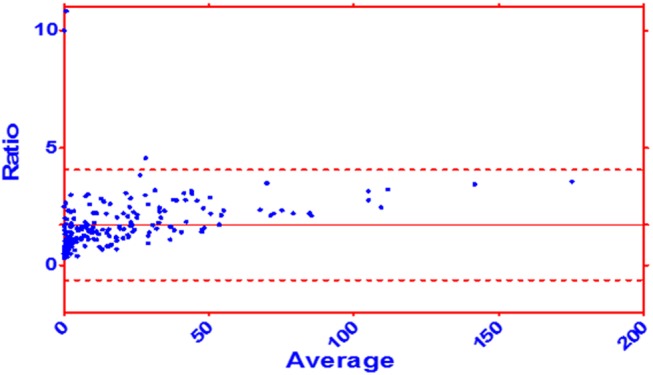
Bland-Altman plot for comparing the od-value with the volumetric acquired lesion volume. Dataset of the day 1 and day 2 combined. Ratio: volumetric volume / od-value. Average: (volumetric volume + od-value) / 2. Mean: 1.722 ± 1.96 SD: -0.626 to 4.071.

To compare the od-values with the volumetric acquired lesion size we generated a Bland-Altman plot with lesion volumes of day 1 and day 2 combined in one plot (Fig 3).

### Analysis of the compiled dataset

Median lesion volume in the compiled dataset (n = 50) was 47.7 ml. The median volume determined with the ABC/2 method was 73.2 ml. Resident 1 provided a systematic underestimation by a median of 32 ml with a Spearmans r = 0.859 (p < 0.01). Resident 2 estimated a median volume of 50 ml. Despite substantial under- and overestimation, his ratings correlated with volumetric results with a Spearmans r = 0.951 (p < 0.01).

**Fig 4:**
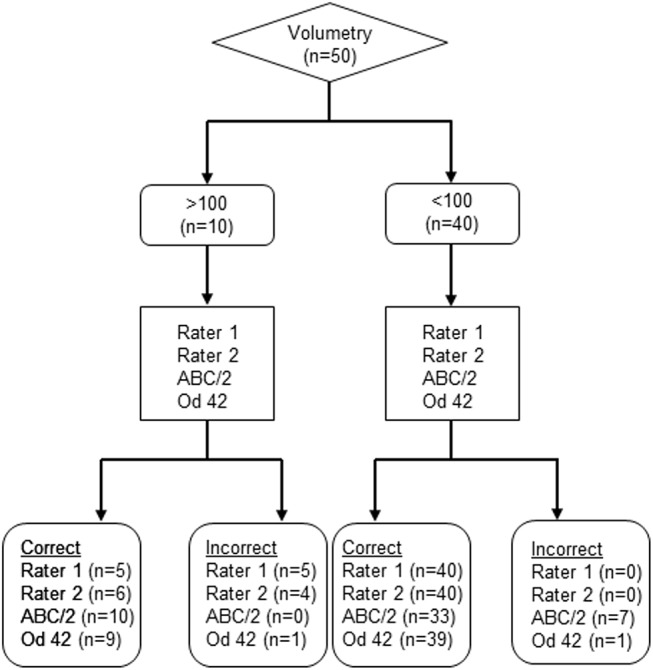
Flow diagram of the > 100 ml threshold. The 50 Patients from the compiled dataset were analyzed. The volumetry was considered gold standard and used as the index test. Then the other methods (Rater 1, Rater 2, ABC/2 and od-value 42) were used to estimate if the volume is > 100 ml, respectively < 100 ml.

**Fig 5:**
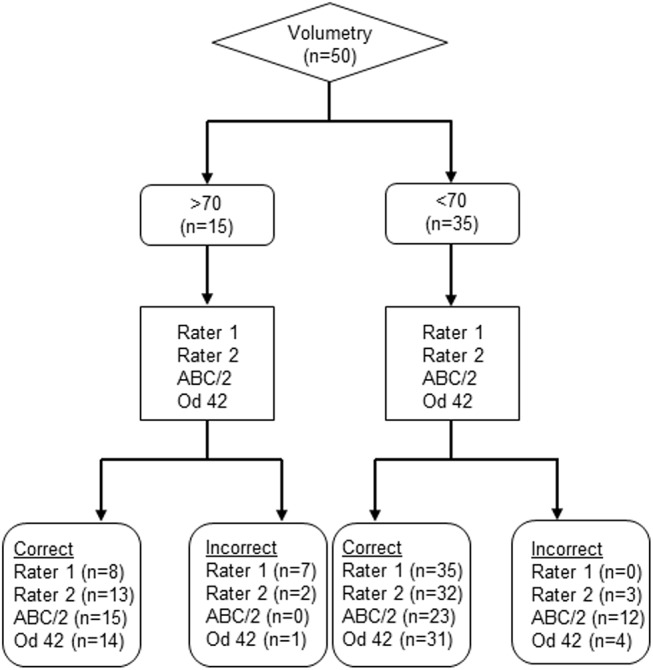
Flow diagram of the > 70 ml threshold. The 50 Patients from the compiled dataset were analyzed. The volumetry was considered gold standard and used as the index test. Then the other methods (Rater 1, Rater 2, ABC/2 and od-value 32) were used to estimate if the volume is > 70 ml, respectively < 70 ml.

All assessments were tested with respect to accurate identification of lesions > 100 ml and > 70 ml. Rater 1 and 2 reached a sensitivity of 50%/ 60% for infarcts > 100 ml, with a 100% specificity. For infarcts > 70 ml sensitivity was 53%/ 87% with a specificity of 100%/ 91%. The ABC/2 method had a sensitivity of 100% for the detection of infarcts > 100 ml and > 70 ml, with a specificity of 66%/ 83%. Identification of infarcts > 100 ml / > 70 ml with od-values had a sensitivity of 90%/ 93% with a specificity of 98%/ 89% (Table 1), (Figs 4 and 5).

**Table 1:** Comparison of visual estimation by two raters, the ABC/2 method and od-values in terms of predicting infarct sizes > 100 and > 70 ml.

	Sensitivity	Specificity	Accuracy	Positive predictive value	Negative predictive value
For predicting lesion size > 100 ml					
**Rater 1**	50	100	90	100	89
**Rater 2**	60	100	92	100	91
**ABC/2**	100	66	76	56	100
**od-value 42**	90	98	96	90	98
For predicting lesion size > 70 ml					
**Rater 1**	53	100	86	100	83
**Rater 2**	87	91	90	81	94
**ABC/2**	100	83	86	59	100
**od-value 42**	93	89	90	78	97

Data for rater 1 and 2 and the ABC/2 method were obtained from the compiled dataset of 50 patients. Od-values are obtained from the day 1 and day 2 lesions of the whole cohort (n = 108 for day 1 and n = 88 for day 2).

## Discussion

The association between acute infarction volume and od-value on DWI can be expressed as quadratic regression equation. Od-values of 42 and higher indicate extended infarction and might indicate patients that are unlikely to benefit from thrombolysis. Applying a 70 ml infarct volume as exclusion criterion corresponds to an od-value of 32.

We observed good correlation between od-values and volumetric measurements of infarct volume on DWI. In the Bland-Altman Plot seen in Fig 3 we compare the od-value with the gold standard: the volumetric approach. We see a broader scattering in the smaller lesion sizes than in the larger lesion sizes. This indicates that the usage of the od-value gets more accurate if the infarct volume gets bigger. An accurate measurement of infarct volume is not required before initiation of thrombolysis, however patients presenting with large infarctions likely to be harmed should be identified [7, 8]. In a previous study comparing sensitivity and infarct extent estimation in CT and DWI, we observed a substantial variability of reading accuracy in resident physicians [2]. A comparable variability was observed in this study: one reader provided systematic underestimation of infarct size, and the other misjudged in both directions. From a clinical perspective this misjudgment caries a risk of withholding therapy as well as putting other patients at risk for symptomatic hemorrhage [8].

In agreement with data reported on the ABC/2 lesion estimation approach [3, 4], we observed a systematic overestimation of the volumes with this method. Excellent sensitivity of ABC/2 was associated with a specificity much lower than for the od-values and even in comparison with visual ratings. In terms of accuracy, the od-value represented the best values for both identifying infarct sizes of > 70 ml and > 100 ml.

Based on the multicentric AXIS trial data the maximal lesion diameter was compared to volumetric assessments in DWI lesion with a median volume of 26 ml [9]. That approach was designed to identify a lesion volume of at least 15 ml to avoid very small infarction in a randomized clinical trial. In contrast we focused on the upper limits of lesion volume to guide thrombolysis and other therapeutic options and therefore selected a sample of cases that included the extremes. The od-value is a simple alternative to visual inspection of lesion volumes. It is easy to obtain and is feasible in an acute clinical setting. Excellent sensitivity of this method for large infarctions together with high specificity increases the chance for guiding treatment towards patients who are likely to benefit. Patients with extended infarction carrying increased risk for secondary deterioration can be identified with od-values > 42 allowing for individualized therapy guidance.

63% of our patients were examined first with a 4.5h time window. The reason for including patients in a later time window was that the rigid time window only applies for iv thrombolysis and does not apply in several clinical trials.

Our study is limited by its monocentric design. The need of obtaining informed consent introduces a bias against very large infarctions as these patients often are unable giving consent. This study is a Retrospective analysis of a prospective trial. Further prospective studies will demonstrate the feasibility of this method in acute clinical setting. Furthermore the exclusion of patients with NIHSS < 8 or with scattered infarcts avoid the application of this method to these patients. Multicenter studies on larger cohorts are necessary to confirm our results in other populations. In addition this study does not provide data about therapeutic utility of the estimation of infarct size based on two diameters on DWI. Thus, only prospective multicenter studies will demonstrate the therapeutic utility of this method in acute clinical setting. The od-value is a promising tool for detecting large infarctions. Whether od-value is likely robust enough to guide patient selection in a randomized trail requires verification with existing data from multicenter studies or from registry to investigate universal validity.

## Supporting Information

S1 STARD Checklist STARD Checklist. (DOC)

S1 Protocol Trial protocol. (DOCX)
