# Supplementary material for: Reliability of Two Diameters Method in Determining Acute Infarct Size. Validation as New Imaging Biomarker
Source: PLoS One. 2015 Oct 8;10(10):e0140065. doi: 10.1371/journal.pone.0140065 (PMC4598169; doi:10.1371/journal.pone.0140065)
Supplement: S1 Protocol — (DOCX) [file pone.0140065.s002.docx]

Application of advice by the local ethic committee for a medical research project which does not include the clinical testing of a pharmaceutical

| 1. Title | „3T Stroke-MRI for examining *mismatch*  at the CSB, Charité Universitätsmedizin Berlin“  Short title: „1000+“ |
| --- | --- |
| 2.ethic committee approval | EA4/026/08 |
| 3. Decisions of other ethic committees | Monocentric, hence no other application of advice to another ethic committee |
| 4. Objective and aims of the study;  Hypotheses, divided into primary and secondary hypotheses, as well as clinical parameters by which those hypotheses should be tested | **Objective:**  Evaluation of acute stroke MRI in patients with suspected stroke/TIA. For this purpose the clinical and economic parameters of patients, examined with an acute stroke MRI (patient group) should be compared to those examined by routine computer tomography (control group).  **Primary hypothesis and aim:**  In acute stroke infarct development can be validated prospectively by means of MRI    **Secundary hypothesis and aim:**  1. By means of acute MRI the supposed diagnosis can promptly be verified or falsified  Assessment of time between admission and final diagnosis and comparison between both groups as well.  2. Treatment optimization and improvement of outcome due to the prompt validation of the suspected diagnosis  Comparison of mRS and NIHSS day 5-7 between both groups  3. Characterization of metabolic changes in ischemic tissue of small subcortical infarcts  Acquisition of diffusion weighted (DWI) and perfusion imaging (PI) with 3nd pass corrected CBF-maps  4. Diffusion tensor imaging in acute stroke (Hamburger DTI-study)  DTI measurement (approval by the ehic committee Hamburg already available)  5. evaluation of structural damage and pathogenesis in patients with transitory ischemic attacs (TIA)  Infarct detection and development by means of DWI  6. fMRI assessment of reorganisation after stroke  ccute and follow-up fMRI  7. DWI lesion comparison, acute and day 5 in male and female patients.  8. Infarct characerization in crytogenic and cardio-embolic infarcts  DWI / FLAIR day 5 infarct location  9. Characterization of cerebrovascular damage in central dizziness and correlation to infarct location  Nystagmography und vestibular testing  10. Characterization and validation of the suspected diagnosis “central vestibular lesion“  DWI / FLAIR |
| 5. ‚Annotation to the importance of the study | The importance of this study concerns its potential use in clinical routine:  By means of acute stroke MRI and prompt validation of the suspected diagnosis, therapy decision and appraisal of benefit / risk are facilitated and improved. A further advantage, compared to standard methods like computer tomography is the lack of radiation exposure or iodinated contrast agents |
| 6. Which of the following assignations apply   1. Medizinproduktegesetz according to § 20 MPG (equipment does not have the declaration of conformity or it does, but is tested by another indication or there are other additional invasive or other incriminatory examinations performed) **or** according to § 23 MPG ? 2. Strahlenschutzverordnung § 23 3. Röntgenverordnung § 28 a 4. Gentechnikgesetz 5. Datenschutzgesetze | a) MR-tomograph and research software are approved medical products  b) no radioactive nuclides  c) no radiation exposure  d) n.a.  e) the assignation for data protection by the county of Berlin are observed (especially secure data storage, no e-mails with data attributable to individuals). Evaluation of patient data will be performed as a “blinded read” |
| 7. If need be: Description and Characterization of the product (equipment for MPG-Studien; please attach appendix) | The examination will be performed at an approved MR tomograph with the appropriate software |
| 8. Substantial results of preclinical studies or reasons why they have not be performed | Mosley first published data in animals on a model of ischemia in 1990. Perfusion MRI was established at the end of the ninties. |
| 9. Substantial content and results of previous studies/applications of the product to be tested in this study | Stroke MRI will be examined in cohort studies since 1995 and is able to identify patients who will benefit of thrombolysis (Köhrmann et al./ Albers et al.). An improved visualization of brainstem infarcts will be achieved by high resolution DWI (Latour et al). MR-based patient selection for thrombolysis was successfully employed for the first time in a multicenter study DIAS/DEDAS (Hacke et al./Furlan et al.). |
| 10. Description of the intended procedures/methods and assumed deviations from the medical routine  (what is routine, what is deviating from routine in the study) | The following examinations, differing from routine, will be performed:   - MRI on admission (T0), the following day T1), day 5-7 (T2) and day 90 (+/-7) (T3) - Recording of mRS and NIHSS at T0, T1, T2 and T3 |
| 11. Assessment and consideration of foreseeable risks and disadvantages compared to the expected benefit for the participants and future diseased persons (benefit / risk assessment) | In rare cases a MRI examination can provoke a stimulation of peripheral nerves and a slight warming of the examined body region. |
| a. Forseeable therapeutic benefit for participants of the study (**individual benefit** for each individual) | The participants benefit is an optimized treatment |
| b. Forseeable medical benefit for future diseased persons **(group benefit)** | Should it be possible to prove that MRI within 24 hours from symptom onset has a significant impact on therapy and outcome, this would influence the acute diagnosis of all future stroke patients |
| c. **Risks** and liability for the participants (all in detail) | Regarding the MRI examination there are risks given the magnetic field regarding metallic particles or implanted electronic devices. Those can be excluded as far as possible by a well-directed medical history / clinical examination  In rare cases there is a warming of body tissue or stimulation of peripheral nerves (even more rare slight paresthesia like prickling or tremor) during the MRI examination  The contrast agent, used for the perfusion protocol, can cause allergic reactions. A renal damage via the MR contrast agent is an extreme rare complication. |
| 12. Action for risk control | Participants will be informed of all possible dangers via a text sheet supplying information regarding the study and MR examination. Additionally they will be questioned for special risks before entering the scanner room. Patients with implanted electronic devices will not be included at all (see exclusion criteria). Adherence to the inclusion and exclusion criteria will be strictly observed. Patients can interrupt the examination at any time (emergency bell, communication with the examiner via intercom)  Um die Lärmbelästigung zu minimieren, tragen die Patienten einen Gehörschutz. |
| 13. Break off criteria | Individually: see No 12 – patients can interrupt the examination at any time (emergency bell, communication with the examiner via intercom). Further reason for interrupting the examination are possible technical problems, which are very rare.  In general: Should there be any medical evidence that the study lack significance or subprojects are published in sufficient number the continuation of this study will be critically tested |
| 14. Participants number, age and gender | It is planed to include about 600 patients / year (> 17 years) in this study |
| 15. Statistical plans, statement and biometric reason for the number of cases and signature of statisticiaN |  |
| 16.  a. Explanation of **in- and exclusion criteria** | **Exclusion criteria:**   - age under 18 years - pregnancy - exclusion criterion for MRI examination, which are not removable metallic pieces (Aneurysma clips, shell splinter, etc.) or implanted elektronic devices (pace maker, pharmaceutical pump, etc.) (see information sheet)   **Inclusion criteria:**   - age: min. 18 years - assumed diagnosis of acute stroke or TIA - informed consent, written or oral in the presence of witnesses |
| b. **Information for participants** (who is granting it verbally and specification, how long is the remaining time between information and consent, otherwise cross reference to the content as appendix) | Participant information will be obtained verbally or in written form by the director of studies or an approbated medical representative directly before the acute examination.  Ahead of the study, from each participant, if he/she is able to, a written informed consent will be obtained according to the prevailing legal assignations  Is the participant not able to do so due to health reasons, a verbal consent in presence of a witness will be obtained. The written informed consent will be obtained later, once the participant is able to. The participant will be informed about the nature, the aim and risks of the study and he will be handed over copies of the information sheet. The patient information sheet explains the nature of the study, the aims and possible risks. Beyond it explains to the participants the detailed requirements and emphasizes that the participation can be cancelled at any time without indication of reasons. Details for accountability are also given. Each participant has the opportunity to talk with and question the director of studies about the information sheet before consenting to the study. |
| c. **Informed consent** (cross reference to content as appendix available) | Participants must agree in written form or verbally in the presence of witnesses (information sheet and informed consent see appendix) |
| d. If need be. **Information and informed consent of the legal representative** (if need be also description how to set up a legal care) | An informed consent by a legally installed custodian is possible |
| 17. Activities to winning participants (notice, zur Gewinnung von Studienteilnehmern (bulletin ?, advertisment? etc.) | Only acute patients from the emergency room and of the CBF are to be included in this study |
| 18. If need be: **Reason for inclusion and explanation of the therapeutic benefit for patients under age and / or for those not able to consent** | Patients who are under age and suffer an ischemic stroke are a rarity and will be included in this study due to the individual advantage of the MRI examination.  Patients not able to consent are generally included in the study, since using MRI over CT radiation exposure and iodinated contrast agents can be avoided.  Stroke MRI is superior to CT in infarct detection and equal with regard to detecting cerebral haemorrhage |
| 19. Relationship between participant and study physician | There is no relationship or dependencies between investigator and participant |
| 20. Declaration of including persons possible dependent on sponsor or study physician | There will be no examinations of volunteers or patients dependent in any kind of a sponsor or study physician |
| 21. Actions how to determine whether a participant participates in several studies at once or ahead of a certain deadline before ending the study | The simultaneous participation of volunteers or patients at an AMG- or MPG study will be excluded by interviewing the participants. If the possibility of a simultaneous study participation cannot be ruled out first, the patient will be examined in the MRI as a start. Does the patient prove to be an AMG- or MPG participant in the end, the data will not be evaluated to exclude a bias |
| 22. If need be: participants reward or compensation (amount, purpose ?) | none |
| 23. If need be: Plan for subsequent treatment and medical supervision of the persons involved at the end of the study | After the end of the study there is the regular patient-centered care by the department of neurology, if necessary |
| 24. If need be: participant insurance (insurance affirmation, insurance conditions, insurance, insurance coverage, length of insurance) | The investigator is covered by the comprehensive general liability |
| 25. If need be: Method of documentation (cross reference to CRF-sheets is possible) | see CRF-sheets attached |
| 26. If need be: Description how to document the health status of healthy affected persons | n.a. |
| 27. If need be: methods to determine, to document and communicate unwanted effects (when, whom and how ??) | All data and events will be documented. Unwanted events (for instance dropout by the participant, technical problems) will be documented and technical problems, if possible, remedied. The forwarding of unwanted events will happen according to general guidelines |
| 28. Approach to protect the confidentiality of the saved data, documents and if need be, the sample, statement how to encode participants data (*no initials and birthdate for encoding, please)* | All compiled data will be stored systematically and digital data will be stored in pseudonymized form. Pseudonymisation will be performed via an encryption code. A list to allocate the patients and the corresponding encryption number is under closure with Dipl.-Phys. Peter Brunecker. The MR images, destined for this study are encrypted too. |
| 29. Declaration for compliance with data privacy act | All data privacy acts will be kept (according to BlnDSG 31.07.2001 and BDSG v. 18.05.2001). Data and results will not be relayed to a third party. |
| 30. Names and addresses of those institutions involved with the study as study centers or study lab, as well as the director of studies and the study physicians | Charité – Universitätsmedizin Berlin, Campus Benjamin Franklin, Hindenburgdamm 30, 12200 Berlin,  CSB Center for Stroke Research Berlin  Charitéplatz 1, 10117 Berlin  Director of studies: Jochen B. Fiebach, Priv.-Doz. Dr.med.  Tel: 0049-30-8445 2275, Fax:0049-30-8445 4264  E-mail: jochen.fiebach@charite.de  Study physicians:   - Gabriele Oepen, Dr. med. - Gerhard Jan Jungehülsing, Dr. med. - Christian Nolte, Dr. med. |
| 31. Statement as for the adequacy of the testing center, especially for the adequacy of the existing means and institutions, as well as the existing staff to realize the clinical testing and experience in realizing similar studies. | Testing center: MR tomograph, department of Neurology, Charité, CBF  MR Siemens Magnetom Trio 3 T  Staff:  PD Dr. med. Jochen B. Fiebach, senior radiologist with the main focus of neuroradiology  Dr. med. Gabriele Oepen, physician of the department of Neurology  Dr. med. Gerhard Jan Jungehülsing, senior neurologist  Dr. med. Christian Nolte, senior neurologist  Claudia Kunze, MTA-R  Dipl.-Phys. Peter Brunecker, data processing  Dipl.-Biol. Kati Jegzentis, study assistent  All staff has broad experience in the organization and realization of clinical studies |
| 32. Agreement on the investigators/main investigators/directors of clinical testing access to data and principles of publication | All data is available in an pseudoanonymized form and will be published by all participating co-workers.  No conclusion of the identity or the participants person can be drawn based upon the publication |
| 33. Information on the studies funding  (see § 263 StGB) | Basic equipment and staff will be provided by the department of Neurology, Charité |
| a. Source of funding (name and location) | IFB-CSB – letter of approval: 20. December 2007: approval until 2013 |
| b. Amount of calculated costs per participant and overall | 800 € per participant; 800.000 € per 1000 patients |
| c. Amount of refunding per participant and overall | - |
